# CAG repeat mosaicism is gene specific in spinocerebellar ataxias

**DOI:** 10.1016/j.ajhg.2024.03.015

**Published:** 2024-04-15

**Authors:** Radhia Kacher, François-Xavier Lejeune, Isabelle David, Susana Boluda, Giulia Coarelli, Sabrina Leclere-Turbant, Anna Heinzmann, Cecilia Marelli, Perrine Charles, Cyril Goizet, Nisha Kabir, Rania Hilab, Ludmila Jornea, Julie Six, Marc Dommergues, Anne-Laure Fauret, Alexis Brice, Sandrine Humbert, Alexandra Durr

**Affiliations:** 1 Sorbonne Université, Paris Brain Institute - ICM, Inserm, CNRS, APHP, Hopital de la Pitié-Salpêtrière, Paris, France; 2 Sorbonne Université, Paris Brain Institute’s Data Analysis Core Facility, Inserm, CNRS, APHP, Hopital de la Pitié-Salpêtrière, Paris, France; 3 Sorbonne Université, Department of Genetics, APHP, Hopital de la Pitié-Salpêtrière, Paris, France; 4 Sorbonne Université, Department of Neuropathology Raymond Escourolle, APHP, Hopital de la Pitié-Salpêtrière, Paris, France; 5 Sorbonne Université, Biobank Neuro-CEB Biological Resource Platform, APHP, Hopital de la Pitié-Salpêtrière, Paris, France; 6 MMDN, Université Montpellier, EPHE, INSERM, Montpellier, France; 7 Expert Center for Neurogenetic Diseases, CHU, Montpellier, France; 8 Université Bordeaux, Equipe « Neurogénétique Translationnelle - NRGEN », INCIA CNRS UMR5287 Université Bordeaux and Centre de Reference Maladies Rares « Neurogénétique », Service de Génétique Médicale, Bordeaux University Hospital (CHU Bordeaux), Bordeaux, France; 9 Sorbonne Université, Service de Gynécologie Obstetrique, APHP, Hopital de la Pitié-Salpêtrière, Paris, France

**Keywords:** spinocerebellar ataxia, SCA, ATXN1, ATXN2, ATXN3, ATXN7, somatic instability, CAG repeat, DNA repair, repeat expansion

## Abstract

Expanded CAG repeats in coding regions of different genes are the most common cause of dominantly inherited spinocerebellar ataxias (SCAs). These repeats are unstable through the germline, and larger repeats lead to earlier onset. We measured somatic expansion in blood samples collected from 30 SCA1, 50 SCA2, 74 SCA3, and 30 SCA7 individuals over a mean interval of 8.5 years, along with postmortem tissues and fetal tissues from SCA1, SCA3, and SCA7 individuals to examine somatic expansion at different stages of life. We showed that somatic mosaicism in the blood increases over time. Expansion levels are significantly different among SCAs and correlate with CAG repeat lengths. The level of expansion is greater in individuals with SCA7 who manifest disease compared to that of those who do not yet display symptoms. Brain tissues from SCA individuals have larger expansions compared to the blood. The cerebellum has the lowest mosaicism among the studied brain regions, along with a high expression of *ATXN*s and DNA repair genes. This was the opposite in cortices, with the highest mosaicism and lower expression of *ATXN*s and DNA repair genes. Fetal cortices did not show repeat instability. This study shows that CAG repeats are increasingly unstable during life in the blood and the brain of SCA individuals, with gene- and tissue-specific patterns.

## Teaser

Large repeated sequences can cause spinocerebellar ataxias (SCAs). They expand in size over time in the blood and the brain, and this instability varies between SCAs and brain regions. This research helps us understand how these genetic changes evolve throughout a person’s life, providing insights for future therapeutic strategies.

## Introduction

The most common cause of spinocerebellar ataxias (SCAs) is a pathological expansion of CAG repeats in coding regions of target genes. These heterozygous repeat expansions lead to dominantly inherited cerebellar ataxias, involving seven genes: *ATXN1* (MIM: 601556) involved in SCA1 (MIM: 164400), *ATXN2* (MIM: 601517) involved in SCA2 (MIM: 183090) and certain forms of Parkinsonism, *ATXN3* (MIM: 607047) involved in SCA3 (MIM: 109150), *CACNA1A* (MIM: 601011) involved in SCA6 (MIM: 183086) and episodic ataxias, *ATXN7* (MIM: 607640) involved in SCA7 (MIM: 164500), *TBP* (MIM: 600075) involved in SCA17 (MIM: 607136).^1^

The size of the CAG repeats inversely correlates with the age at onset (AO); larger repeats tend to manifest earlier and exhibit greater clinical severity and shorter survival.^2^ However, a large phenotypic variability exists among individuals not solely explained by the size of the expansion inherited at conception.^3^ As in Huntington disease (MIM: 143100), the pathological CAG repeats in SCAs are present at conception, but the symptoms only begin during adulthood, suggesting that age-related factors could be involved.^4^^,^^5^

Meiotic instability reflects the fact that expansions are unstable during transmission, which accounts for anticipation in age AO, but instability is also somatic.^6^ This phenomenon was studied in Huntington disease, where somatic expansion of the CAG repeats increases during the life of individuals, especially in the brain.^7^^,^^8^^,^^9^ One explanation could be that DNA repair processes contribute to an increased repeat size during life, particularly in post-mitotic cells (i.e., neurons). In SCAs and Huntington disease, polymorphisms in DNA repair genes are associated to variability in residual age AO, not explained by the size of the repeat expansion.^10^^,^^11^^,^^12^

In this study, we used one of the standard technique available in diagnosis to study somatic expansion across four dominant ataxias: SCA1, SCA2, SCA3, and SCA7. We used longitudinal analysis of somatic expansion to have an overview of the increase in CAG repeat at different stages in life, including fetal brain and postmortem brain samples.

## Material and methods

### Sample collection

#### Brain samples

Collection of brain samples (Table 1) was performed as part of a national program of Brain Donation for Research (National Neuro-CEB Brain Bank, GIE Neuro-CEB BB-0033-00011). Autopsies were authorized according to French current regulation, and the next of kin authorized the use of samples for research. For each case, one hemisphere was frozen at −80°C, and the contralateral hemisphere was fixed in 4% buffered formalin. In the formalin fixed hemisphere, 1 cm coronal sections were performed, and sampling of representative regions of the neocortex, subcortical nuclei (striatum, thalamus, globus pallidus, subthalamic nucleus), brain stem (midbrain, pons, medulla oblongata), cerebellum, and spinal cord (when available) was performed (Table S6). The samples were embedded in paraffin, cut at 3 μm thickness, and stained with Hematoxylin-Eosin (H&E). In predetermined samples, a Luxol Fast Blue with H&E and immunohistochemical staining was performed for ubiquitin (rabbit polyclonal, Dako, 1/500), p-62 (clone 3/P62 lck ligand, mouse monoclonal, BD Biosciences, 1/500), 1C2 (clone 5TF1-1C2, 1/4,000), Aβ (clone 6F/3D, mouse monoclonal, Agilent, 1/200), phospho-tau (pS202,pT205) (clone AT8, mouse monoclonal, ThermoFischer, 1/500), TDP43 (rabbit polyclonal, Proteintech, 1/1,000), and α-synuclein (clone 5G4, mouse monoclonal, Millipore, 1/4,000).

**Table 1:** Cohort description, postmortem brains

**ID**	**Pathology**	**Sex**	**AD**	**CAG**	**Expansion index**																				
					**Cerebellum (CAG)**	**Dentate**	**Pons**	**Pons body**	**Pons head**	**Olive**	**Medulla oblongata**	**Midbrain**	**Midbrain tegmentum**	**Substantia nigra**	**Thalamus**	**Pallidum**	**Caudate**	**Amygdala**	**Primary motor cortex**	**Primary visual cortex**	**Frontal cortex**	**Blood (years from AD)**	**Blood (years from AD)**	**Blood (years from AD)**	**Blood (years from AD)**
1	SCA1	M	42	55	1.26 (54)	N/A	N/A	N/A	N/A	N/A	N/A	N/A	N/A	N/A	3.42	3.22	3.04	2.45	N/A	N/A	3.52	N/A	N/A	N/A	N/A
2	SCA1	F	57	49	0.31	N/A	N/A	N/A	N/A	N/A	N/A	N/A	N/A	N/A	N/A	N/A	N/A	N/A	N/A	N/A	N/A	N/A	N/A	N/A	N/A
3	SCA1	M	50	49	0.36	N/A	N/A	N/A	N/A	N/A	N/A	N/A	N/A	N/A	N/A	N/A	N/A	N/A	N/A	N/A	N/A	N/A	N/A	N/A	N/A
4	SCA2	M	35	47	0.37	0.49	1.46	N/A	N/A	N/A	1.28	0.39	N/A	N/A	1.30	1.31	N/A	1.80	1.69	N/A	2.18	0.79 (−17)	0.94 (−18)	1.20 (−2)	1.20^a^ (−1)
5	SCA3	F	68	73	0.76 (72)	N/A	N/A	N/A	N/A	N/A	N/A	N/A	N/A	N/A	1.70	1.43	N/A	N/A	1.79	N/A	2.01	0.91 (−12)	0.92^a^ (−3)	N/A	N/A
6	SCA3	M	42	78	0.77 (77)	N/A	N/A	1.67	1.66	1.59	1.25	N/A	1.25	1.57	1.67	1.33	N/A	N/A	1.73	N/A	1.74	N/A	N/A	N/A	N/A
7	SCA3	F	56	74	0.59 (73)	N/A	N/A	1.64	1.65	1.31	1.18	N/A	1.37	1.39	N/A	N/A	N/A	N/A	1.39	N/A	1.60	0.61 (−17)	0.65 (−11)	N/A	N/A
8	SCA3	F	72	70	0.42 (69)	N/A	N/A	1.83	1.87	1.75	N/A	N/A	1.70	1.65	N/A	N/A	N/A	N/A	1.75	N/A	1.81	N/A	N/A	N/A	N/A
9	SCA7	M	55	42	0.52	N/A	1.56	N/A	N/A	1.72	N/A	N/A	N/A	N/A	N/A	N/A	N/A	N/A	1.38	1.72	1.55	N/A	N/A	N/A	N/A
10	SCA7	M	56	42	0.56	N/A	N/A	2.28	2.01	1.86	N/A	N/A	1.81	2.23	1.76	N/A	N/A	N/A	1.60	2.71	1.86	1.89 (−25)	2.85 (−12)	3.81^a^ (−5)	N/A
11	SCA1	M	42	54	N/A	N/A	N/A	N/A	N/A	N/A	N/A	N/A	N/A	N/A	N/A	N/A	N/A	N/A	N/A	N/A	N/A	1.10^a^ (−15)	N/A	N/A	N/A
Mean SCA1			50	51	0.64	N/A	N/A	N/A	N/A	N/A	N/A	N/A	N/A	N/A	N/A	N/A	N/A	N/A	N/A	N/A	N/A	N/A	N/A	N/A	N/A
SD SCA1			8	3,5	0.54	N/A	N/A	N/A	N/A	N/A	N/A	N/A	N/A	N/A	N/A	N/A	N/A	N/A	N/A	N/A	N/A	N/A	N/A	N/A	N/A
Mean SCA3			60	74	0.64	N/A	N/A	1.72	1.73	1.55	1.22	N/A	1.44	1.53	1.69	1.38	N/A	N/A	1.67	N/A	1.79	0.76	0.78	N/A	N/A
SD SCA3			14	3,3	0.17	N/A	N/A	0.10	0.13	0.22	0.04	N/A	0.23	0.13	0.02	0.07	N/A	N/A	0.19	N/A	0.17	0.21	0.19	N/A	N/A
Mean SCA7			56	42	0.54	N/A	1.56	2.28	2.01	1.79	N/A	N/A	1.81	2.23	1.76	N/A	N/A	N/A	1.49	2.22	1.70	1.89	2.85	3.81	N/A
SD SCA7			1	0	0.02	N/A	N/A	N/A	N/A	0.10	N/A	N/A	N/A	N/A	N/A	N/A	N/A	N/A	0.15	0.70	0.22	N/A	N/A	N/A	N/A

Descriptive data on the postmortem brain cohort. ID, identification number; AD, age at death; CAG, modal CAG at diagnosis; cerebellum (CAG), EI in the cerebellum (with main CAG peak in the cerebellum if different from modal CAG); blood (years from AD), blood sample (−years before death); SD, standard deviation.

a Blood samples used for qPCR analysis. SCA1: *n* = 3 (49, 49, 54 CAG); SCA2: n = 1 (47 CAG); SCA3: *n* = 4 (73, 78, 74, 70 CAG); SCA7: *n* = 2 (42, 42 CAG); SCA1 blood: age sample 27, age at death 42.

#### Fetal samples

Following prenatal testing, parents can request termination of the pregnancy performed by manual vacuum aspiration under general anesthesia. The termination occurs usually at gestational week 13 (GW13). We used standard obstetric protocols in accordance with the French guidelines for clinical practice. Prenatal visits and psychological support were provided for all couples participating, as standard practice, and no additional visits were planned due to participation in this study. The women signed an informed consent during a prenatal visit agreeing to the collection of fetal tissue following the eventual termination of the pregnancy. The study complied with all relevant ethical regulations, with approval from the French Agency of Biomedicine (no. PFS17-001; January 24, 2017).

#### Longitudinal study

All tested subjects were offered long-term follow up, and they signed an informed consent prior to clinical examination and interview. We determined age AO based on self-reported age and examination by a neurologist. We followed SCA individuals in the SPATAX network with written informed consent according to the French legislation SPATAX RBM01-29/BIOMOV APH210069 Sud-est IV. 2021-A00989-32. Inclusion criteria were an SCA phenotype, defined as the presence of ataxia and a CAG repeat expansion in the associated gene: *ATXN1*, *ATXN2*, *ATXN3*, and *ATXN7*.

### DNA extraction

Postmortem brains and fetal tissues were frozen and stored at −80°C until DNA extraction. DNA was extracted using the Maxwell RSC Tissue DNA Kit (Promega), according to the manufacturer’s instruction. We measured DNA yields using a NanoDrop 8000 spectrophotometer (Thermo Scientific).

### Determination of the CAG repeat length

Amplification of the CAG repeat in *ATXN1*, *ATXN2*, *ATXN3*, and *ATXN7* was performed as follows: in a final volume of 25 μL, each PCR reaction contained 200 μM of each deoxyribonucleotide triphosphate (dNTP), 5 pmol of each primer (see table in supplemental material), 200 ng of genomic DNA and 1X PCR-Buffer, 1X Q-Solution, and 1 unit of Taq DNA polymerase (solution stock at 5 unit/μL, Qiagen). The PCR steps are as follows: denaturation for 10 min at 96°C; 35 cycles of 1 min of denaturation at 96°C, 1 min of annealing at 65°C, 1 min of extension at 72°C; and final extension for 7 min at 72°C. Each amplification product was mixed with Hi-Di Formamide and Genescan-400HD Rox size standard (Applied Biosystems). Fragments were separated on an Applied Biosystems 3730XL DNA Analyzer. We scored alleles with the Gene Mapper software v5.0 (Applied Biosystems). We used primers coupled to fluorescent probes for each *ATXN* (see supplemental methods, primers sequence—determination of CAG length by PCR).

### Analysis of the CAG repeat length and expansion index

We used the Gene Mapper software v5.0 (Applied Biosystems) to analyze the CAG repeat expansions. For an individual, the PCR products peak around a main signal representing the main CAG size. Signal before this peak includes PCR stutter inherent to the assay that can bias the true biological variation in CAG repeat size. Therefore, we did not consider the peaks before the main CAG peak. PCR products at greater lengths represent somatically expanded CAG repeats present in each tissue. From the Gene Mapper sample plot view, we exported a data table for each sample containing the following information: sample name, called CAG allele, peak size in base pair (bp), peak height, area under the peak, and data point/scan number of the highest point of the peak. Based on the main expanded CAG peak size, we used an internal standard to assign a main CAG length to each sample specific for each gene and on a per plate basis. We used peak heights to quantify expansion levels from Gene Mapper traces. To calculate the proportion of expanded products for each sample, we normalized the heights of the expanded peaks to the sum of all peak height multiplied by the peak position, giving a relative proportion compared to the main CAG. We applied a relative threshold of 0.03 of the main peak and excluded from analysis peaks falling below this threshold. We selected this threshold based on the additional peaks in fetal tissues that were low in intensity but clearly distinguishable from background by the software. Finally, we summed the values for each peak to generate an expansion index (EI) (Figure S1^9^^,^^13^). To consider the longitudinal aspect of the study, for EI calculation, the same modal CAG was used for all traces from the same individual.

### Statistical analysis

We conducted all statistical analyses using R version 4.3.1 (R Development Core Team, 2023; https://www.R-project.org/), and we generated plots with the ggplot2 R package^14^ (v3.4.3) and ComplexHeatmap^15^ (v2.16.0) R packages. The level of statistical significance was set at two-sided values of *p* < 0.05 for all tests.

### Descriptive statistics

We reported descriptive statistics for individuals with demographics and disease characteristics (sex, age, EI) determined at each visit that included blood collection. We defined age AO as the onset of motor signs, as defined by the person, or first neurological exam at which they were symptomatic, whichever was earlier. Clinical evolution of signs is evaluated using the Scale for the Assessment and Rating of Ataxia (SARA) score, a clinical scale to assess the severity of cerebellar ataxia, which is composed of eight items. It ranges from zero to 40, zero indicating absence of ataxia and 40 indicating the most severe degree of ataxia.^16^ Manifest ataxia is then defined when individuals have a score greater than 3.5. We calculated a severity score, the ratio of SARA score divided by disease duration. We summarized the data as n (number of available values) and mean ± standard deviation (SD) for quantitative variables, frequency counts and percentages for categorical variables.

### Relationship between somatic EI and age

We studied EI evolution over time using a linear mixed-effects model (LMM). The model included age, (CAG)n, the disease group and their interaction terms as fixed effects, and a random intercept effect on the individual identifier. The significance for the main interaction effects was evaluated using Type II Wald chi-square tests. The LMM was fitted using the function lmer in the lme4 package^17^ (v1.1-34). Type II Wald chi-square tests were performed using the function Anova in the car package (v3.1-2). The regression slopes of EI over age were estimated for each group using the emtrends function of the emmeans package (v1.8.8) in order to reflect the overall evolution of EI in each disease. These slopes were then tested against zero and compared between the groups using Kenward-Roger approximation of degrees of freedom and Tukey’s method for comparing a family of four estimates. To allow comparison of the groups, the variables Age and (CAG)n were mean centered by group on the characteristics at first visit prior to modeling. By doing so, the comparison of slopes was interpretable in terms of “mean individuals,” i.e., between individuals virtually associated with the average characteristics of their respective groups.

### Regression analysis of disease characteristics with CAG repeat and somatic expansion measures

For all individuals, to investigate whether the tendency to expand increases over time, we derived individual rates of change of the EI by simple linear regressions of the EI values on the ages at successive visits. From regression lines, both slope and intercept coefficients were extracted to obtain, respectively, the value of expansion rate (ER, which corresponds to the additional level of expansion per year) and an EI intercept, which would correspond to a theoretical baseline value of the EI at birth.

### Relationship between EI/ER with germline CAG repeat length and severity

We studied the relationship between the somatic expansions and the CAG repeat length by linear regressions, one regression for each disease group. We then assessed the strength of association with the Pearson’s correlation coefficient (r), and the *p* value of the regression slope.

We performed linear regressions to model the values of severity (SARA scores corrected for disease duration) within each disease group. To account for the influence of age and the CAG length, we used the residual values of ER obtained by linear regressions performed on (CAG)n within each disease group. We then used the Pearson’s correlation coefficient (r), and the *p* value of the regression slope for association testing.

### Relationship between somatic expansion measures and disease status

With the presence of preataxic individuals at first visit (SCA1: 8 [26.7%], SCA2: 11 [22.0%], SCA3: 15 [20.3%], SCA7: 9 [30.0%]), we were able to compare the levels of EI at first visit and the values of ER between the preataxic and ataxic stages for each group. To adjust for age and (CAG)n, comparisons were based on the residual values of EI obtained by linear regressions performed on age and (CAG)n and residual values of ER obtained by linear regressions performed on (CAG)n within each disease group. To account for a potential nonlinear relationship between CAG and age, we tested for a quadratic effect of age that did not show any significant effect. Wilcoxon rank-sum tests were then used for the two-group comparisons of EI and ER residuals.

### RNA extraction and quantitative real-time PCR

Postmortem brains and fetal tissues were rapidly frozen and stored at −80°C until RNA extraction. We extracted the RNA from brain tissues using the Maxwell RSC simplyRNA Tissue Kit and from blood samples using the Maxwell RSC simplyRNA Cells Kit, according to the manufacturer’s instruction (Promega). We measured RNA yields using a NanoDrop 8000 spectrophotometer (Thermo Scientific). RNA quality was also assessed with the RNA integrity number using the TapeStation system (Agilent). Reverse transcription was performed with the RevertAid First Strand cDNA synthesis kit (Thermo Scientific) with 100 ng of RNA for each sample. Quantitative PCR was performed using the Biomark HD system (Fluidigm Corporation) with the 48.48 dynamic array IFC for gene expression and SsoFast EvaGreen Supermix, according to the manufacturer’s protocol. See supplemental methods for primers sequences. We used *PPIA* expression to normalize cDNA amount. *PPIA* was tested on cerebellum and cortical control tissues to validate the stability of cycles. Differential expression was calculated using the 2-ΔΔCt method^18^; each individual was normalized to their respective cerebellum. A complementary analysis was included by extracting data from the Genotype-Tissue Expression (GTEx) portal (https://gtexportal.org/home/), where RNA sequencing was performed on flash-frozen non-diseased tissues.

## Results

### Determination of somatic expansion index in SCA cohorts

The participation of 184 SCA individuals allowed us to analyze the clinical variability regarding the progression and severity of the phenotype over 8.5 years on average. We collected biological samples from 30 SCA1, 50 SCA2, 74 SCA3, and 30 SCA7 individuals at different points in life (Tables S1–S5), as well as 3 fetuses (SCA1, SCA3, and SCA7) and 11 postmortem brains (Table 1). For all samples, we calculated an EI based on a PCR followed by fragment sizing to identify the peaks corresponding to the number of CAG repeats, or (CAG)n.^13^^,^^19^ The expanded allele has a characteristic profile where the highest peak provides the CAG repeat size used for diagnosis. Additional peaks are present after the main peak, revealing the various repeat lengths existing in each tissue, and thus reflecting the degree of somatic expansion. The fluorescence intensity of each peak reflects the proportion of cells bearing each CAG repeat size. Since the polymerase slippage during PCR can bias the proportion of peaks to the left of the reference peak, we analyzed only those to the right to calculate the EI (see material and methods; Figure S1). An EI of zero indicates no expansion beyond the inherited allele; an increased index indicates further expansion of the CAG repeat above the main peak.

### Somatic instability of the CAG repeats increases during the life of SCA individuals

**Figure 1:**
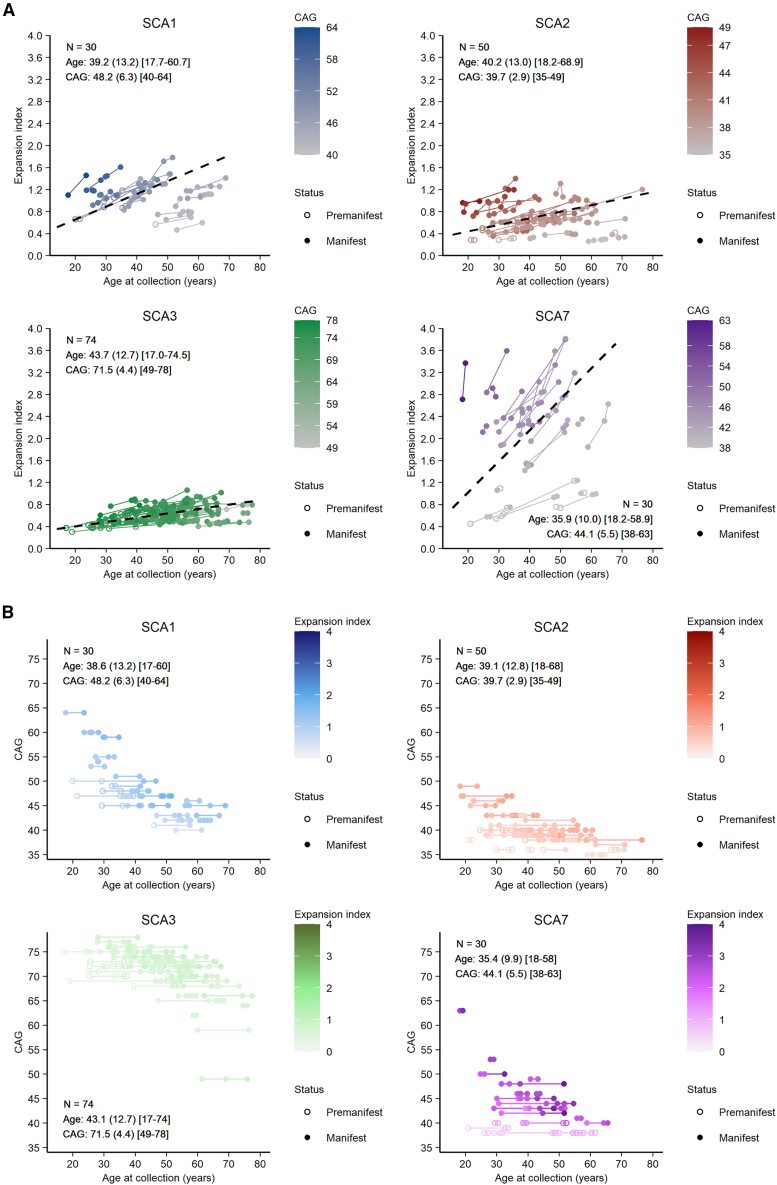
Somatic expansion of the CAG repeats increases in the blood of SCA1, SCA2, SCA3, and SCA7 individuals (A) Individual trajectories of the EI across visits (up to eight visits) for each SCA group (one panel per group). The color gradient represents the range of CAG repeats in each group with darker shading indicating higher (CAG)n. Summary data are individuals' average characteristics (age and CAG repeats) at the first visit. For each value, the disease status is indicated with an empty circle (premanifest; before disease onset) or a filled circle (manifest; after disease onset). The dashed black regression line represents the progression of the EI as a function of age corresponding to the mean CAG(n) of the group, with the slope estimated using the emtrends function (emmeans R package) based on a mixed-effects model fit. For the four groups, the EI increases with age, with the most striking increase for SCA7 individuals, and the slowest increase for SCA3 individuals. (B) Longitudinal data plotted by (CAG)n, the color gradient represents the range of EI with darker shading indicating higher EI.

Blood samples were collected during clinical visits at different ages, with two to three visits for most individuals (Tables S1–S5), up to eight visits, taken on average 8.5 years apart between the first and the last visit (range: 1–31 years, Figure 1). SCA7 individuals had the highest EI values with the highest increase over time whereas SCA3 individuals had the lowest EI values with the smallest variations over time (Figure 1A).

We used a linear mixed model to longitudinally characterize the relationship of EI with age, SCA group, and expanded CAG repeat size. There was a significant three-way interaction effect showing that EI increases with age, and CAG repeat sizes for all pathologies (Wald χ^2^ [degrees of freedom = 3] = 108.1, *p* < 2.2e-16). By setting the number of CAGs to the average number of repetitions obtained for each SCA group, we observed a mean annual EI increase with significant slope values (mean CAG/mean annual increase ±standard error; SCA1: 48.2/0.023 ± 0.003, SCA2: 39.7/0.012 ± 0.002, SCA3: 71.5/0.008 ± 0.002, SCA7: 44.1/0.055 ± 0.003; all *p* < 0.0001; dashed black lines in Figure 1A). Comparing these slopes, we found that the EI increased significantly more in SCA7 compared to SCA1, SCA2, and SCA3 (all *p* < 0.0001) and significantly more in SCA1 compared to SCA2 (*p* = 0.02) and SCA3 (*p* < 0.0001), while the increase was indistinguishable between SCA2 and SCA3 (*p* = 0.30).

Previous studies in Huntington disease showed that with larger repeat expansions, the somatic expansion was greater.^7^^,^^9^ Similarly, Pearson’s (r) correlation coefficient showed a significant linear relationship between EI and (CAG)n in the blood for SCA1 (r = 0.48, *p* = 7.6e-3), SCA2 (r = 0.55, *p* = 4.0e-5), and SCA7 (r = 0.78, *p* = 4.7e-7) (Figure S2A). Although *ATXN3* has larger CAG repeats in absolute terms (around 70 CAG repeats), this did not influence the propensity to expand at a given time (r = 0.04, *p* = 0.72) (Figures 1B and S2A). To estimate the progression of expansion for each individual, we established an ER that corresponds to the ratio of the EI on the time between visits. The ER correlates with (CAG)n for SCA7 (r = 0.56, *p* = 1.2e-3) and for SCA3 individuals but to a lesser degree (r = 0.27, *p* = 0.018, Figure S2B).

### EI correlates with the clinical progression for SCA1 and SCA7 individuals

**Figure 2:**
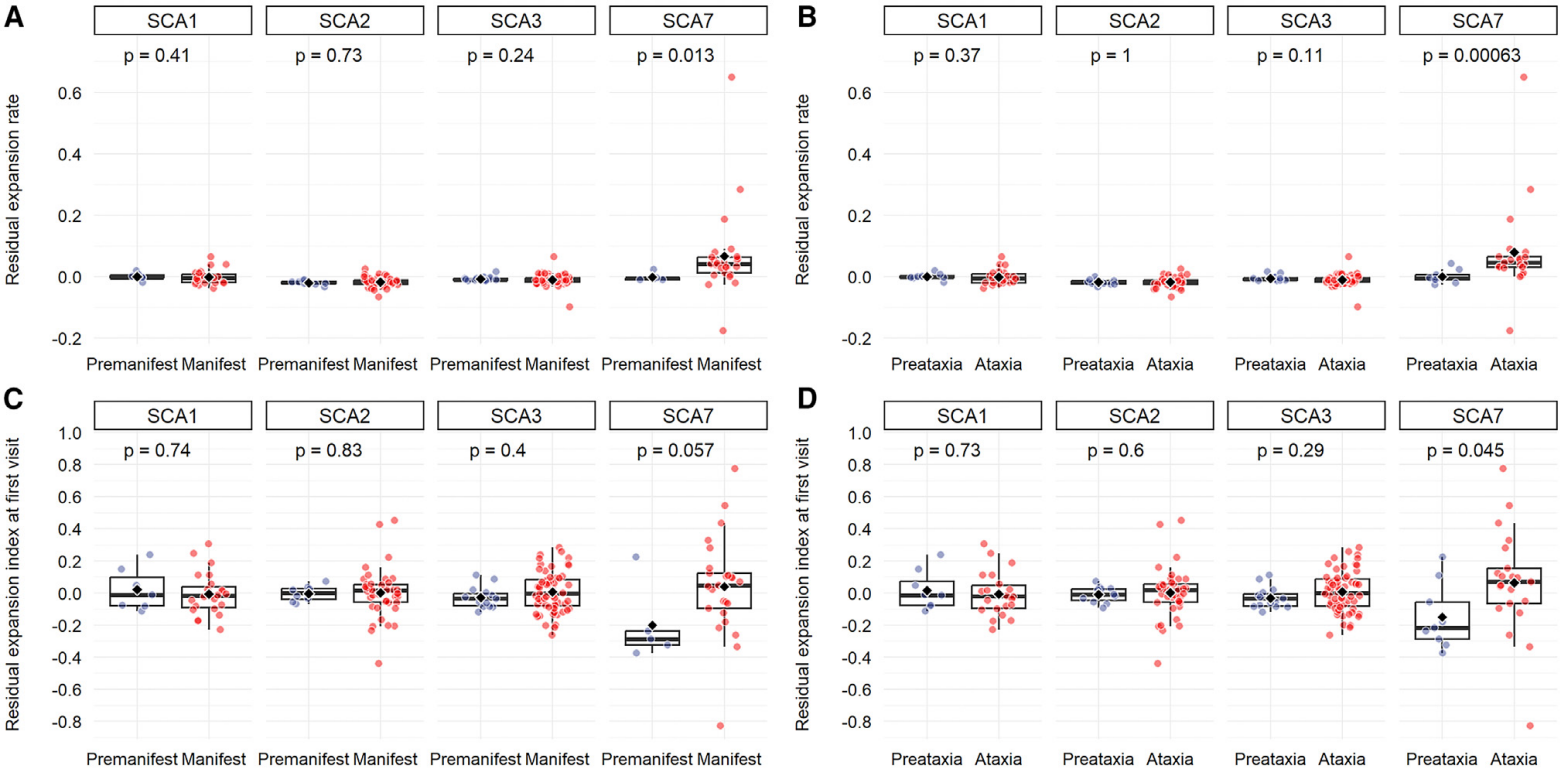
Residual ER correlates with disease status for SCA7 individuals Boxplots showing the distribution of the residual ER (i.e., corrected for [CAG]n) (A) between the individuals’ groups classified as premanifest (no clinical signs) and manifest (SARA >3.5 and/or pyramidal signs at examination), (B) or only based on the SARA score (preataxic <3.5, ataxic >3.5). Boxplots showing the distribution of the residual EI (i.e., corrected for age and (CAG)n) (C) between the individuals’ groups classified as premanifest (no clinical signs) and manifest (SARA >3.5 and/or pyramidal signs at examination) (D) or only based on the SARA score (preataxic <3.5, ataxic >3.5). *p* values of the two-sided Wilcoxon’s rank-sum tests are shown at the top of each plot.

We analyzed the correlation between the degree of the somatic expansion and the status of the individual either premanifest (no clinical signs) or manifest (SARA >3.5 and/or pyramidal signs at examination). The residual ER (increased expansion per year corrected for CAG effect) is significantly different between premanifest and manifest SCA7 individuals (*p* = 0.013), but not for the other groups (Figure 2A). The same observation was done considering only the SARA score, preataxic SCA7 individuals (SARA <3.5) have a lower residual ER compared to ataxic individuals (*p* = 0.00063, Figure 2B). With residual EI at first visit (EI corrected for CAG and age effects), we see a trend of higher EI in manifest individuals (*p* = 0.057) and a significantly higher EI for ataxic individuals (*p* = 0.045) again for the SCA7 group (Figures 2C and 2D).

Then, to account for the disease duration, we calculated a severity score, which is the ratio of the SARA score divided by the disease duration. The residual ER correlates with severity at second visit for SCA1 individuals (r = 0.6, *p* = 0.011). At first visit, we observe a trend between residual rate and severity but it is not significant (p = 0.07), this could be due to fewer available clinical data at first visit (*n* = 11 for the first visit and *n* = 17 for the second visit, Figures S3A and S3B).

### The CAG repeat is somatically unstable in the postmortem brain of SCA individuals

**Figure 3:**
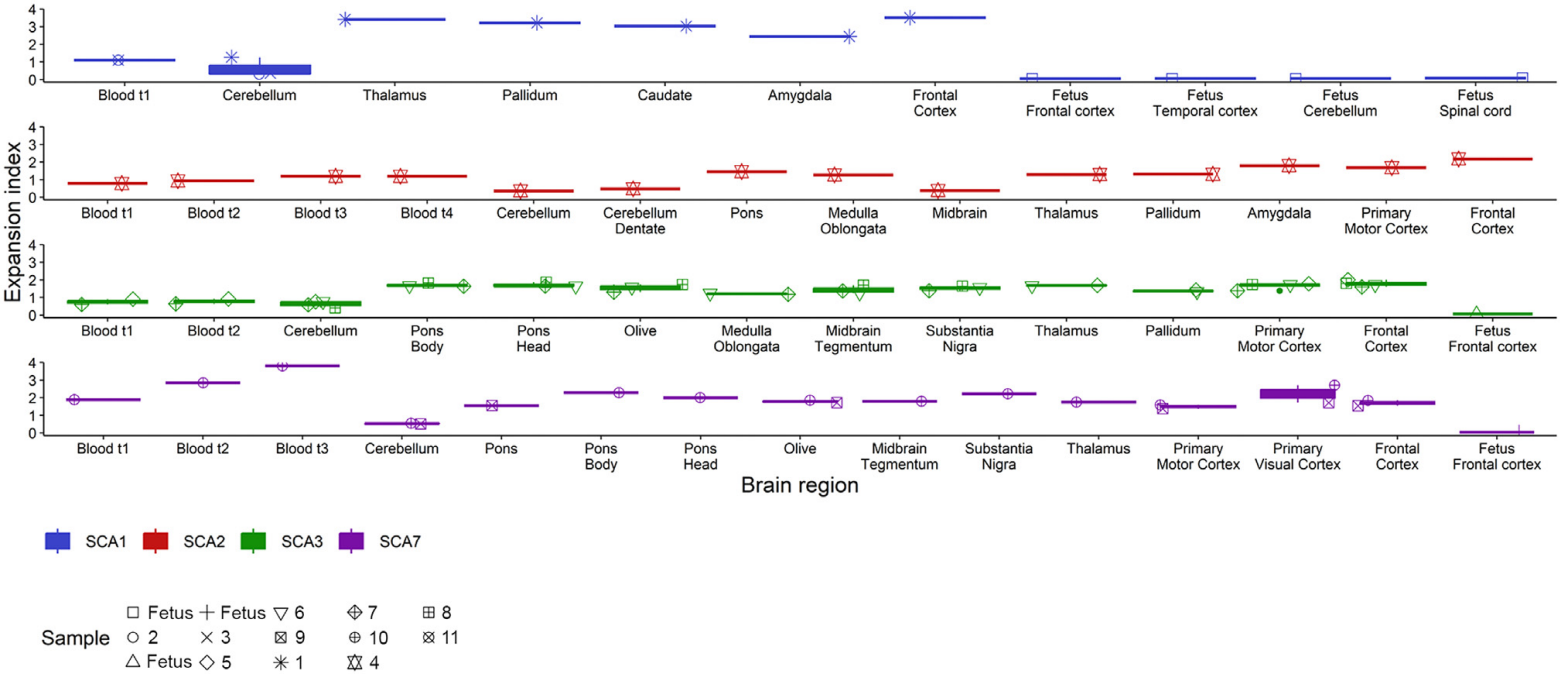
The CAG repeat is somatically unstable in the postmortem brain of SCA1, SCA2, SCA3, and SCA7 individuals Boxplots of the EI measured in different postmortem brain structures. When only one sample was available, the value is plotted as one horizontal line instead of a boxplot. Fetal brain samples were available for SCA1, SCA3, and SCA7 and have the most stable repeat compared to the adult brain. Pathology and number for each individual is indicated at the bottom (matching data for each individual is available in Table 1). t1, first visit; t2, second visit; t3, third visit; t4, fourth visit.

We analyzed EI in the most affected postmortem brain regions (Figure 3; Tables 1 and S6). For SCA1 (individual 1, Table 1) we did not have the corresponding blood sample, yet if we compare to an individual from the longitudinal cohort with a similar profile (54 CAG, age 42 at death: individual 11, Table 1), the EI 15 years prior to death is 1.10, lower than in the brain. For SCA2 (individual 4, Table 1), EI one year before death was 1.20, lower than in most analyzed brain structures except the cerebellum and the midbrain. We analyzed two SCA3 individuals with corresponding blood samples (individuals 5 and 7, Table 1); for both, EI at the last visit was lower in blood, except in the cerebellum where EI was the lowest. Interestingly, for SCA7 (individual 10, Table 1), EI was higher in the blood with an EI of 3.81 five years before death; we also observed a gain of one (CAG)n at each visit (brain: 42 CAG; blood first, second, and third visit: 42, 43, and 44; interval between visits: 13 years, 7 years).

EI was the lowest in the cerebellum in all SCAs (mean EI in SCA1: 0.64 ± 0.54, SCA2: 0.37, SCA3: 0.64 ± 0.17, SCA7: 0.54 ± 0.02). Not only is the EI lower in the cerebellum, but the main CAG peak also has one less repeat compared to the other brain regions for SCA3 individuals (73, 78, 74, and 70 [CAG]n vs. 72, 77, 73, and 69 in the cerebellum) and the SCA1 individual (55 [CAG]n versus 54 in the cerebellum). In contrast, all SCAs showed the highest EI in the cortex (frontal cortex for SCA1: 3.52, SCA2: 2.18, SCA3: 1.79 ± 0.17, and primary visual cortex for SCA7: 2.22 ± 0.70).

**Figure 4:**
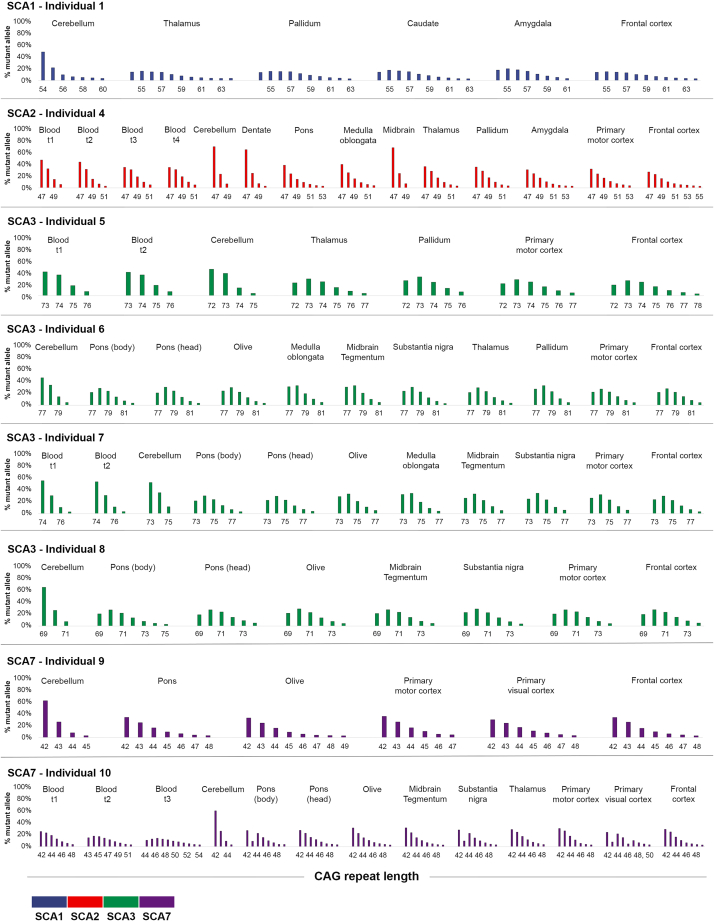
Accumulation of larger CAG repeats in postmortem brains of SCA1, SCA2, SCA3, and SCA7 individuals We ascertained the “% mutant alleles” (as in Figure S1) from the peak heights from PCR profiles obtained on GeneMapper. Each additional peak corresponds to an additional CAG repeat. Pathology and number for each individual is indicated on top of each graph (matching data for each individual is available in Table 1). t1, first visit; t2, second visit; t3, third visit; t4, fourth visit.

We then analyzed the number of additional CAG repeats by measuring the percentage of the sample containing a given CAG repeat (Figure 4). The SCA1 individual with 54 CAG has the largest additional CAG repeats, ranging from six in the cerebellum to 10 in the thalamus and frontal cortex. The most unstable region for the SCA2 individual is the frontal cortex with eight additional CAGs, whereas the cerebellum and the midbrain only have two. SCA3 individuals have on average the lowest number of additional CAG repeats; interestingly these numbers are consistent for a given region among the four individuals, therefore it does not appear to be influenced by the initial length of the modal CAG for these SCA3 cases. As seen with the EI, the blood has the largest accumulation of repeats for the SCA7 individual. Yet, in SCA7 brain regions, the repeat is largely unstable especially in the pons for individual number 9 and in the primary visual cortex for individual number 10. Finally, for all individuals, the structure tested with the least repeats is the cerebellum. The cerebral cortex is the region with the least atrophy (Table S6) and the highest expansion, whereas the cerebellum had lower expansion with higher atrophy. Yet, other regions with atrophy showed high expansion like the pons in SCA3 and the olive in SCA7 (Table S6; Figure 4). The SCA2 individual had more cell loss in the dentate compared to the Purkinje cell layer (Table S6), but these two regions had similar levels of CAG expansion (Figure 4).

### No somatic expansion in fetal tissues in SCA1, SCA3, and SCA7

**Figure 5:**
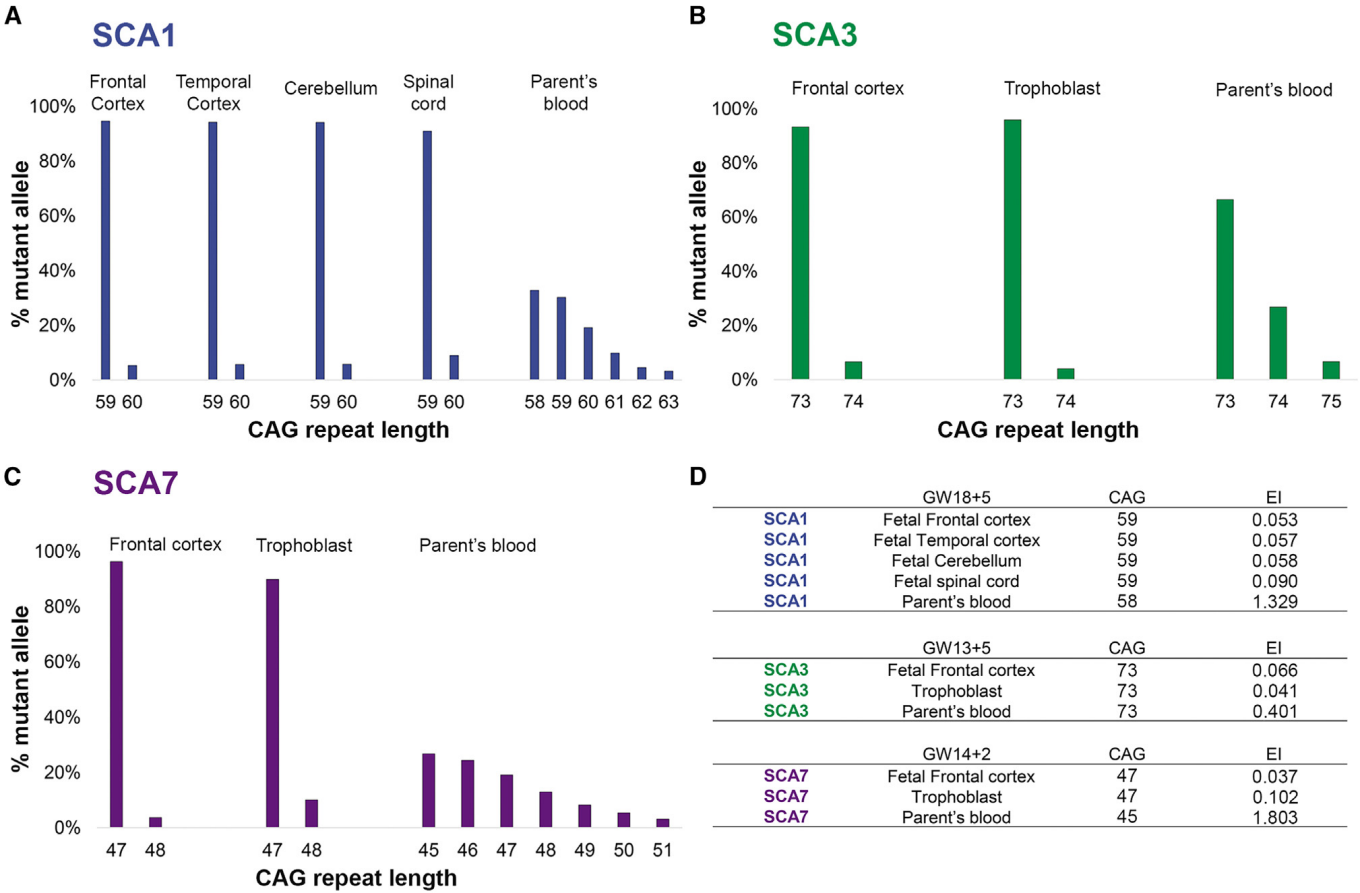
The CAG repeat is somatically stable in the developing brain of SCA1, SCA3, and SCA7 fetus (A–C) (A) Comparison of mosaicism in four CNS region from a SCA1 fetus at 18 gestational weeks and blood of the parent; (B) the fetal cortex, trophoblast, and blood of the parent for an SCA3 fetus at 13 gestational weeks, (C) and a SCA7 fetus at 14 gestational weeks. We ascertained the “% mutant alleles” (as in Figure S1) from the peak heights from PCR profiles obtained on GeneMapper. (D) Detailed EI values with the corresponding modal CAG are indicated in a table.

Although the adult cortex carries significant mosaicism, the fetal cortex showed very low EI (Figure 5D): 0.066 for the SCA3 fetus at 13 gestational weeks (GW13); 0.037 for the SCA7 fetus at GW14; and 0.053, 0.057, 0.058, and 0.090 for the SCA1 fetus at GW18 (frontal cortex, temporal cortex, cerebellum, and spinal cord, respectively).

These indexes were in the same range as those from trophoblast tissues that were analyzed for prenatal diagnosis (tissues collected between GW11 and GW12), with an EI of 0.041 for the SCA3 trophoblast and 0.102 for the SCA7 trophoblast (Figures 5B and 5C). Yet, blood samples taken from parents (premanifest, not part of the longitudinal cohort) had somatic expansions, with an EI of 0.401 for the SCA3 parent (modal CAG: 73 at 25 years old) and an EI of 1.803 for the SCA7 parent (modal CAG: 45 at 30 years old). However, the SCA7 fetus has a larger modal CAG of 47, when the parent was diagnosed with 45 CAG repeats. Of note, the SCA7 parent sample was collected 3 years before the pregnancy. Similarly, the SCA1 parents had a higher EI of 1.329 measured in the blood (at 26 years old, two years before pregnancy). The SCA1 fetus had a larger modal CAG of 59, when the parent was diagnosed with 58 CAG repeats (Figure 5A). To visualize these differences, we plotted somatic mosaicism in fetal tissues and parents’ blood. There is a higher percentage of larger repeats in the parental blood compared to the fetal brain tissue.

### Expression of *ATXN* and DNA repair genes

Among the described causes of CAG repeat instability, the role of DNA repair genes was highlighted, especially in genome-wide association studies.^11^^,^^12^ The main processes that could trigger repair are DNA replication, DNA maintenance, and the transcription of the target gene, *ATXN* in this case.

**Figure 6:**
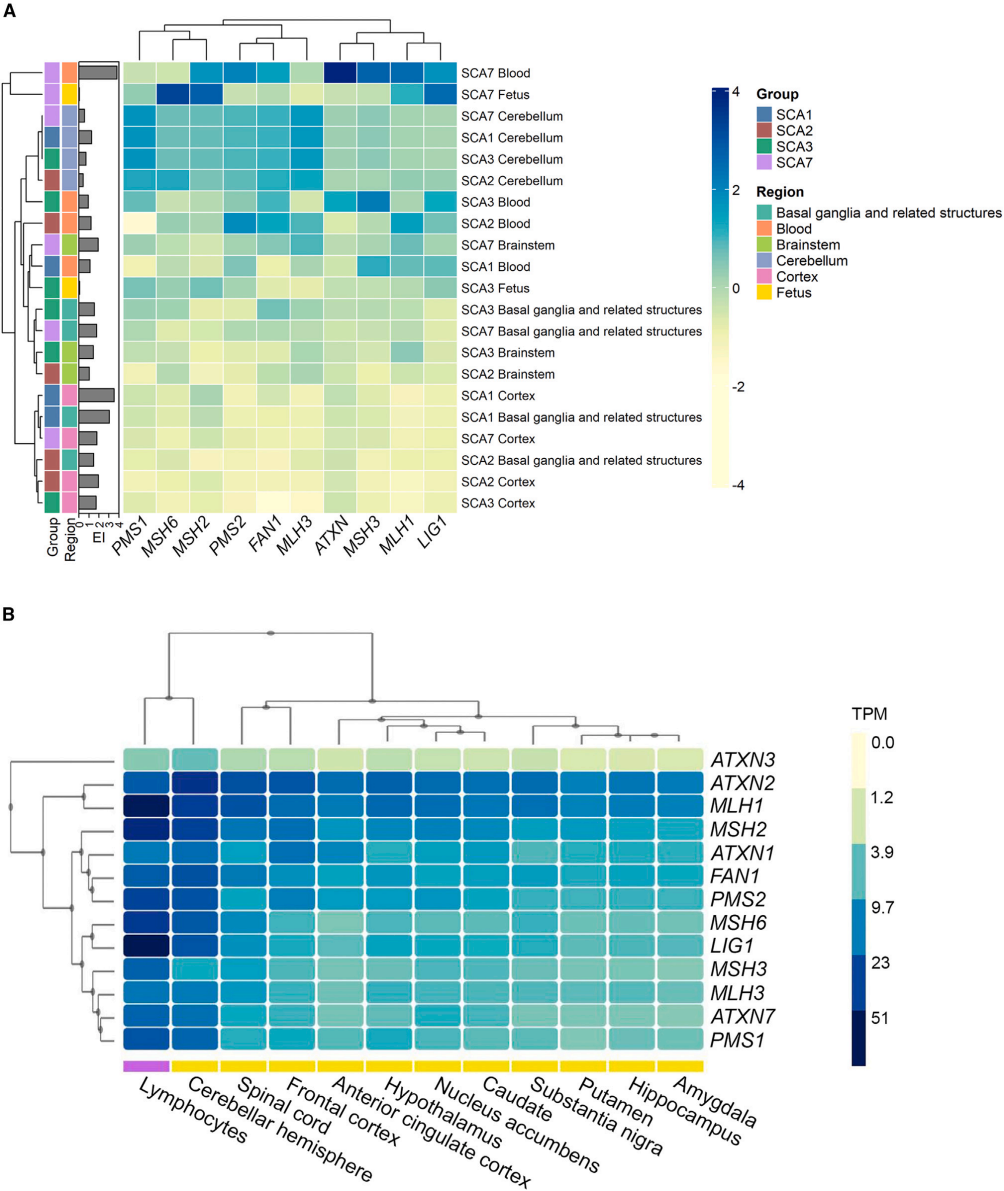
Expression of DNA repair genes is tissue specific (A) DNA repair genes and *ATXN* expression was measured by real-time qPCR and plotted in a heatmap alongside EI on the left. For each disease, the corresponding *ATXN* was analyzed (*ATXN1* for SCA1, *ATXN2* for SCA2, *ATXN3* for SCA3, *ATXN7* for SCA7). Brain regions were grouped as follows: basal ganglia and related structures (amygdala, caudate, pallidum, thalamus), blood, cerebellum, fetus, cortex (frontal cortex, motor cortex, visual cortex), and brainstem (midbrain, substantia nigra, pons, olive, medulla oblongata). For visualization, the qPCR values were scaled to zero mean and unit variance (relative values from 4 to −4). SCA1: *n* = 1 (cerebellum: *n* = 3), SCA2: *n* = 1, SCA3: *n* = 3, SCA7: *n* = 2. (B) Expression levels obtained from the GTEx portal. RNA sequencing was performed on flash-frozen, non-diseased tissues; TPM, transcripts per kilo base million).

*ATXN* levels were comparable in most brain regions except for the cerebellum where it is higher for *ATXN1*, *2*, *3*, and *7* (Figure 6A: SCA individuals; Figure 6B: non affected individuals). *ATXN3* and *ATXN7* have their highest expression in the blood. High level of *ATXN* corresponded to high levels of EI only for the SCA7 individuals in the blood sample. In the fetal brain, both *ATXN3* and *ATXN7* have comparable levels to those measured in the postmortem brain. For one SCA7 individual, the highest EI was found in the blood, with the highest *ATXN7* expression.

We measured the expression of the main DNA repair genes previously described as modifiers in Huntington disease: *MLH1* (MIM: 120436), *MLH3* (MIM: 604395), *MSH2* (MIM: 609309), *MSH3* (MIM: 600887), *MSH6* (MIM: 600678), *PSM1* (MIM: 600258), *PSM2* (MIM: 600259), *LIG1* (MIM: 126391), and *FAN1* (MIM: 613534).^10^^,^^11^^,^^12^^,^^20^ When we compared the global expression of those genes, the heatmap regrouped the samples by brain structures rather than pathology (Figures 6A and S4). Especially, the expression pattern of DNA repair genes in the cerebellum is similar in all SCAs, with high expression of *PMS1*, *MLH3*, *FAN1*, *PMS2*, *MSH6*, and *MSH2*. Blood samples from SCA3 and SCA7 individuals have the highest expression of DNA repair genes, especially *MSH3*, *MLH1*, *PMS2*, *FAN1*, and *LIG1*. In the fetal tissues, *LIG1*, *MSH2*, and *MSH6* have the highest expression (Figures 6A and S4).

To further explore the expression of these genes, we extracted the expression levels of *ATXN*s and DNA repair genes from the GTEx portal (Figure 6B). In this database, RNA sequencing was performed on control tissues from individuals without expanded CAGs. In these control cases, we also observed that the brain region with higher expression of *ATXN*s and DNA repair genes was the cerebellum, and the lymphocytes had a higher level of expression compared to that in the brain.

## Discussion

This longitudinal study of somatic expansion of the CAG repeats in four SCAs, revealed that somatic mosaicism in the blood and brain is gene specific: SCA7 being the most unstable repeat and SCA3 being the most stable. In our previous study on somatic expansion in Huntington disease, we tested the hypothesis of the Kaplan model.^21^ This mathematical model states that the progression of repeat diseases is determined by the rate of somatic expansion in relevant cells, with a hypothetical pathogenic threshold of CAG repeat. We validated this idea in the context of Huntington disease,^9^ yet in this study on SCAs, we show several differences according to the affected gene and the affected brain areas. However, we have no clear explanation for the striking differences in somatic instability between SCAs.

The common feature of Huntington disease and SCAs is the global progression of somatic mosaicism with age. At the beginning of life, somatic expansion is negligible; the repeat is the most stable in fetal tissues in Huntington disease,^9^ SCA1, SCA3, and SCA7. These data strengthen the idea that in the active developmental mitotic environment, DNA repair mechanisms allow control of CAG expansion. Interestingly, instability was shown to be inversely correlated to cell cycle and mitosis whereas it was positively correlated to neurotransmitter activity and metabolism.^13^ The SCA1 and SCA7 individuals showed an increased number of (CAG)n between the parent and the fetus, confirming the germline instability in SCA7 and SCA1.^22^^,^^23^^,^^24^ Anticipation explained by instability of the repeat size was described in all SCAs, with greater increase associated with paternal transmission in SCA1, 2, and 7.^22^^,^^23^^,^^25^^,^^26^^,^^27^^,^^28^

During the life of SCA individuals, somatic expansions accumulate at different levels depending on the affected gene. Residual ER correlates with the severity score for SCA1 individuals. Further confirmation in a complementary SCA1 cohort would be interesting to determine if CAG expansion is a good marker to follow disease severity. SCA7 ataxic individuals have a higher level of expansion compared to premanifest individuals. Until now, the accumulation of the disease protein has been used as a biomarker in Huntington disease or SCA3;^29^^,^^30^ the EI described here in SCA7 could be used to distinguish affected individuals for trial enrollment purposes. A study in a larger group would strengthen these data.

We found that the EI increased with larger (CAG)n repeat. Yet, the level of expansion is lower for SCA3 individuals. Considering that *ATXN3* has the largest repeat size threshold, these data highlight the importance of the genomic context to understand the CAG repeat instability. For instance, the toxicity of the expanded poly-glutamine (at the protein level) correlates with the GC content of DNA flanking sequence (at the genomic level).^31^ Interruptions in the CAG sequence are known to modify the stability of the repeat expansion and disease outcome (Figure S5). We did not analyze this parameter; however, it would be important to consider it to have a full picture of the genetic context. For SCA1 individuals, the absence of CAT interruptions is associated with earlier disease onset (Figure S5).^32^ Moreover, loss of CTG interruptions in *ATXN1* increases instability, with expansion occurring more often at the 5′ side of the interruption.^33^ CAA interruptions in *ATXN2* cf. stability and are present in large normal alleles^34^; CAA interruptions in CAG expansions of this gene cause pure Parkinsonism without cerebellar signs (Figure S5).^35^ In a drosophila study, CAA interruptions in *ATXN3* reduce toxicity.^36^ At the 5′ of *ATXN3* repeat, CGG rather than GGG is associated with disease (Figure S5).^37^ A study in an *ATXN7* mouse model showed that the 3′ region must be present to observe somatic expansion.^38^

Finally, the level of expansion was higher in the postmortem brain for SCA1, SCA2, and SCA3 but not for SCA7 where it was the highest in the blood. The cerebellum had the lowest EI in all SCAs. A few other studies found that the cerebellum is the most stable region in SCA1 individuals^19^^,^^25^ and mouse model,^39^ in one SCA2 family,^40^ and in SCA3 individuals.^41^ In addition, in Huntington disease, where the cerebellum is not a region primarily affected, the CAG repeat expansion on the Huntingtin gene is the most stable in the cerebellum.^19^^,^^42^ The atrophy of the cerebellum cannot explain by itself the lack of expansion; other atrophied regions, such as the pons and the olive, showed high levels of expansion. Among the sample tested, the most unstable region was the cortex. Considering the sample size, a confirmation cohort would strengthen these data.

To explore the mechanism at the origin of the differences between brain structures, we analyzed the levels of expression of *ATXN*s. Indeed, growing evidence associates transcription levels with repeat instability. In a SCA1 mouse model, the elimination of transcription-coupled nucleotide excision repair dramatically reduces CAG repeat instability, specifically in the brain.^43^ In SCA3 individuals, variants in three transcription-coupled repair genes are associated with CAG instability.^44^ In our cohort, *ATXN7* and *ATXN3* are more expressed in the blood compared to the brain. The common observation in our samples was that the *ATXN*s were highly expressed in the cerebellum, contrasting with the low level of expansion. Previous studies suggest the same tendencies; in SCA7 individuals, a northern blot analysis compared brain structures showing higher levels of *ATXN7* in the cerebellum.^24^ Similarly, in an RNA-sequencing database established on the general population (proteinatlas.org), *ATXN3* and *ATXN7* are more expressed in the blood compared to the brain.^45^

Since transcription-induced instability requires mismatch repair elements, we also analyzed the expression of DNA repair genes relevant in CAG repeat diseases. A previous study described DNA repair genes as modifiers of age AO in a combined study on Huntington disease and SCAs.^10^ This was confirmed in another cohort of SCA3 individuals, with an earlier onset of 2.44 years for individuals with G/G genotype at rs3512 (*FAN1*) and a protective effect with the C allele.^46^ Expression of DNA repair genes was high in the cerebellum, compared to other brain structures. Specifically, the highest expression was with *PMS1*, *MLH3*, and cross-link repair *FAN1*. This result was surprising considering the studies on *MSH2* or *MSH3*, which when knocked down reduced repeat contractions, and *MLH1* or *PMS2*, which when depleted increased contraction frequency.^47^ However, *FAN1* is associated with a stabilization of somatic expansion,^7^ and loss of *FAN1* function lead to increased expansion.^48^^,^^49^

Like in Huntington disease, we would have expected a stronger mosaicism in the most affected brain regions in SCAs, such as the cerebellum, but especially in SCA1 and SCA7, that was not the case. It could be coupled to a specific pattern of DNA repair gene expression. The specific mechanism involved in DNA repair in the cerebellum needs to be elucidated. We would need an analysis at the single-cell level to understand the cell specificity of somatic expansion in the cerebellum.

This work describes the progression of CAG repeat size in SCAs over long durations. It especially highlights the importance of identifying the relevant DNA repair mechanism to establish a therapeutic strategy targeting the repeat expansion, which is the basis of pathogenicity.

## Data and code availability

The datasets supporting the current study have not been deposited in a public repository because they include sensitive clinical data but are available from the corresponding author on request.

## Supplemental information

Document S1. Figures S1–S5, Tables S1–S6, and supplemental methods

Document S2. Article plus supplemental information
