# Supplementary material for: CAG repeat mosaicism is gene specific in spinocerebellar ataxias
Source: Am J Hum Genet. 2024 Apr 15;111(5):913–26. doi: 10.1016/j.ajhg.2024.03.015 (PMC11080609; doi:10.1016/j.ajhg.2024.03.015)
Supplement: Document S1. Figures S1–S5, Tables S1–S6, and supplemental methods [file mmc1.pdf]

The American Journal of Human Genetics, Volume *111*

Supplemental information

CAG repeat mosaicism is gene specific in spinocerebellar ataxias

Radhia Kacher, François-Xavier Lejeune, Isabelle David, Susana Boluda, Giulia Coarelli, Sabrina Leclere-Turbant, Anna Heinzmann, Cecilia Marelli, Perrine Charles, Cyril Goizet, Nisha Kabir, Rania Hilab, Ludmila Jornea, Julie Six, Marc Dommergues, Anne-Laure Fauret, Alexis Brice, Sandrine Humbert, and Alexandra Durr

Figure S1

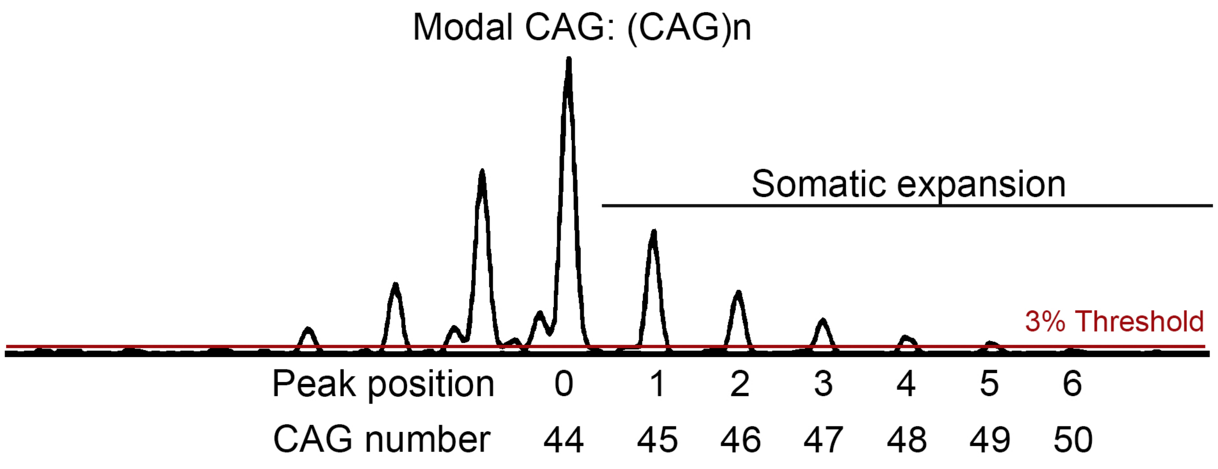

Expansion index = Sum of all peak values
Peak value = (peak height ÷ sum of all peak heights) x peak position

% mutant allele = peak height ÷ sum of all peak heights

Figure S1. PCR profile and expansion index determination. Method for calculating the expansion index (EI) and the percentage of mutant alleles. We considered peaks to represent somatic expansions only if they reached a threshold of at least 3% of the height of the main peak.

Figure S2

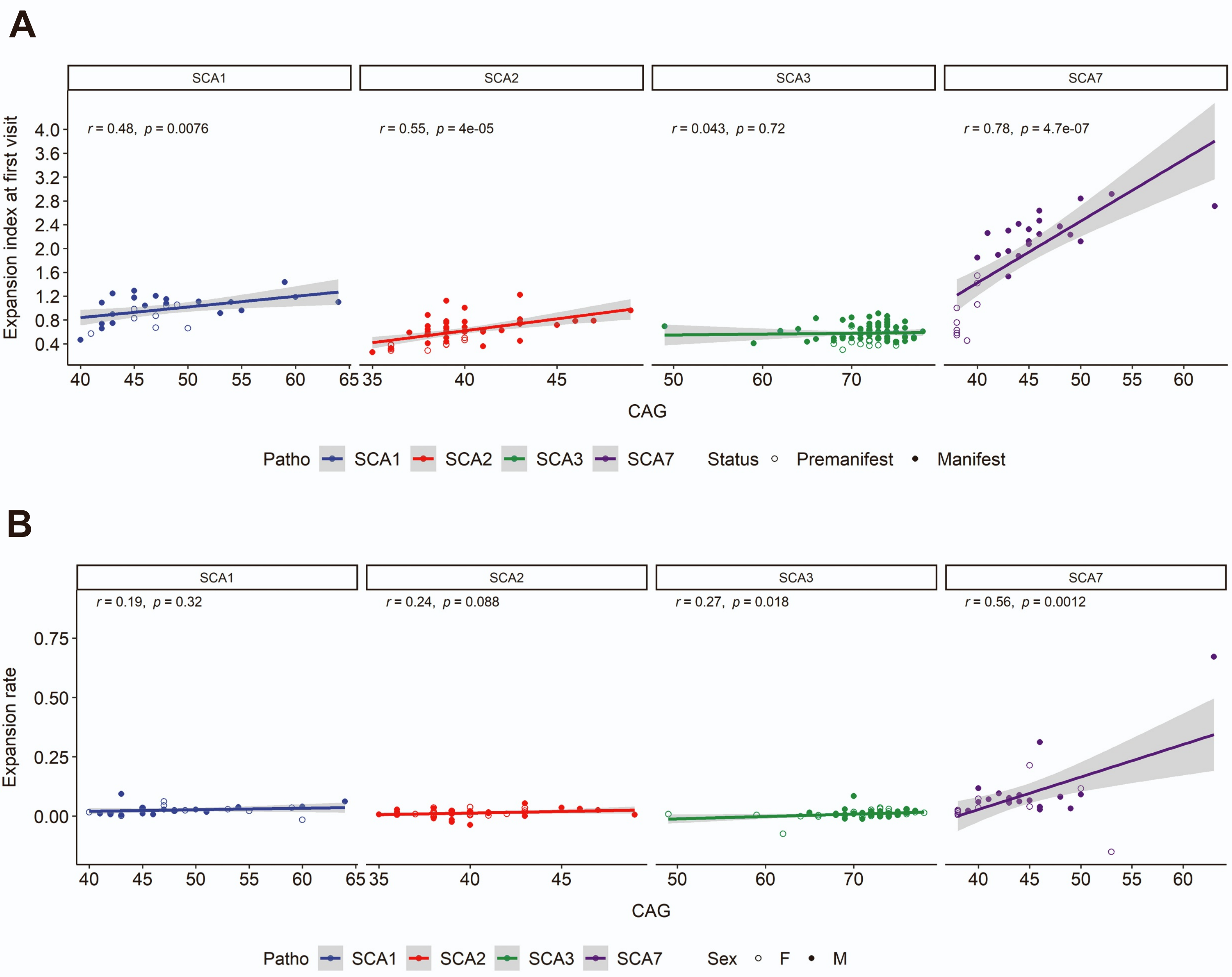
A
SCA1
SCA2
SCA3
SCA7
r = 0.48, p = 0.0076
r = 0.55, p = 4e-05
r = 0.043, p = 0.72
r = 0.78, p = 4.7e-07
Expansion index at first visit
CAG
Patho SCA1 SCA2 SCA3 SCA7
Status Premanifest Manifest
B
SCA1
SCA2
SCA3
SCA7
r = 0.19, p = 0.32
r = 0.24, p = 0.088
r = 0.27, p = 0.018
r = 0.56, p = 0.0012
Expansion rate
CAG
Patho SCA1 SCA2 SCA3 SCA7
Sex F M

Figure S2. Increased expansion index correlates with larger CAG repeat length for SCA1, SCA2 and SCA7 whereas expansion rate correlates with CAG repeat length for SCA3 and SCA7. Scatter plots showing modal CAG repeat length correlation to the expansion index measured at the first visit (A) or to the expansion rate (B). (A) The status is indicated with an empty circle (Premanifest) or filled circle (Manifest). (B) The sex is indicated with an empty circle (F, woman) or filled circle (M, man). p-values (*p*) and correlation coefficient (*r*) for the linear regressions are above each plot. Colored curves denote estimated linear regression of the data with 95% confidence intervals shaded in gray.

Figure S3

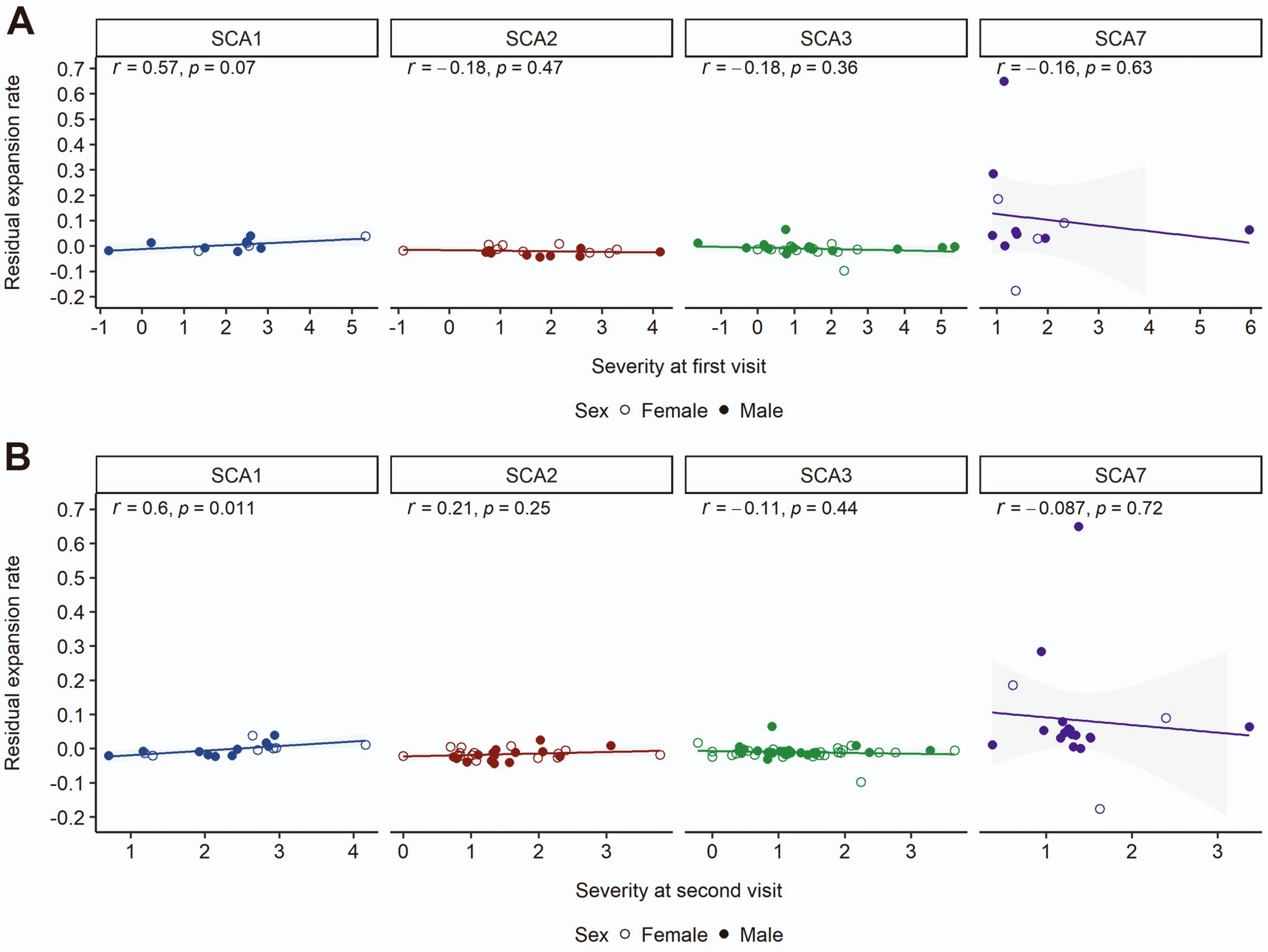
A
SCA1
SCA2
SCA3
SCA7
r = 0.57, p = 0.07
r = −0.18, p = 0.47
r = −0.18, p = 0.36
r = −0.16, p = 0.63
Residual expansion rate
Severity at first visit
Sex ○ Female ● Male
B
SCA1
SCA2
SCA3
SCA7
r = 0.6, p = 0.011
r = 0.21, p = 0.25
r = −0.11, p = 0.44
r = −0.087, p = 0.72
Residual expansion rate
Severity at second visit
Sex ○ Female ● Male

Figure S3. Residual expansion rate correlates with severity for SCA1 individuals. Scatter plots showing residual Expansion Rate (ER corrected for CAG effect) against severity at first (A) and second visit (B). p values (*p*) and correlation coefficient (*r*) for the linear regressions are above each plot. Colored curves denote estimated linear regression of the data with 95% confidence intervals shaded in gray. The sex is indicated with an empty circle (F, woman) or filled circle (M, man).

Figure S4

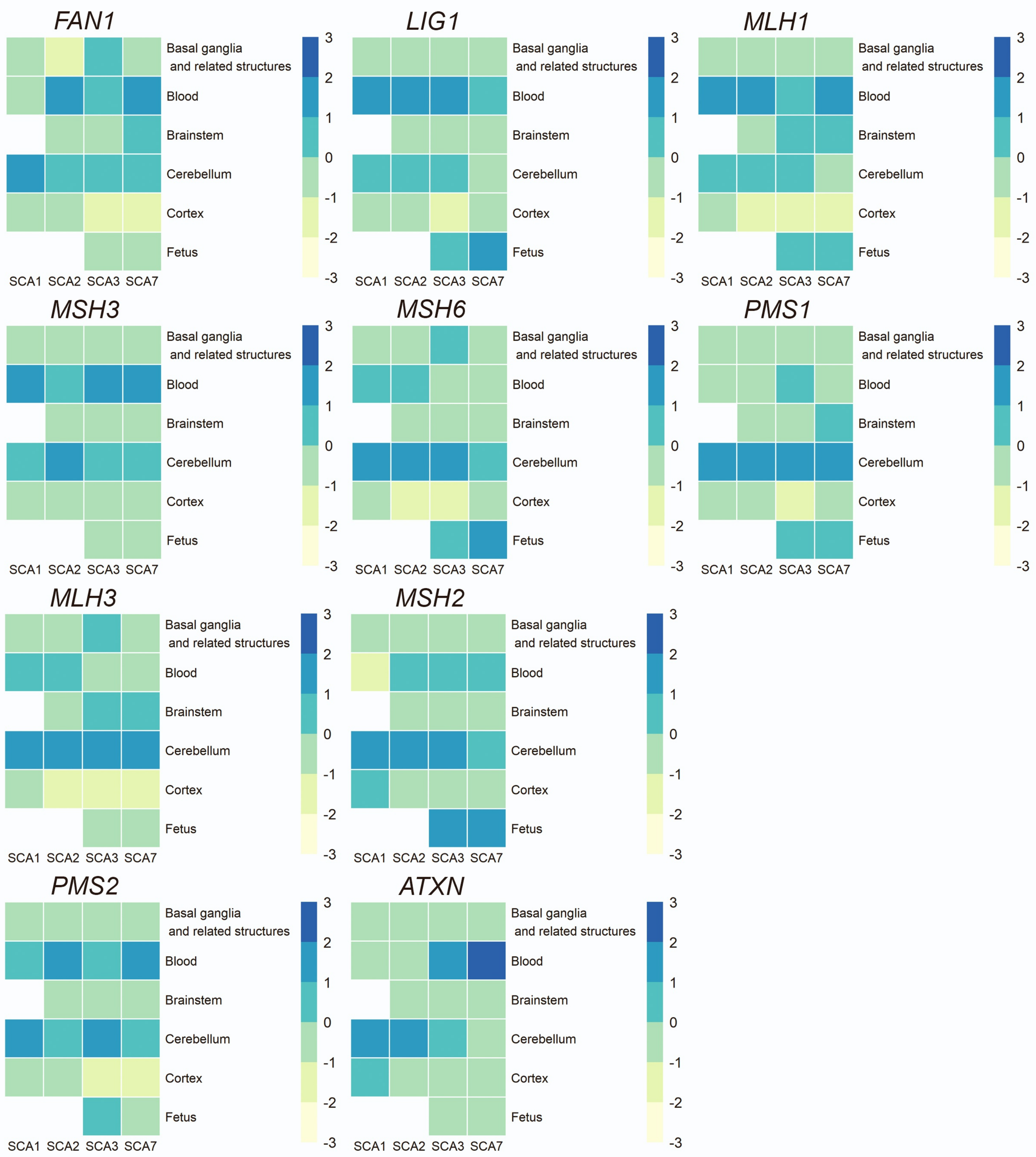
FAN1
LIG1
MLH1
MSH3
MSH6
PMS1
MLH3
MSH2
PMS2
ATXN
Basal ganglia and related structures
Blood
Brainstem
Cerebellum
Cortex
Fetus
SCA1
SCA2
SCA3
SCA7
3
2
1
0
-1
-2
-3

Figure S4. Differential expression of DNA repair genes in different brain regions in SCAs. DNA repair genes and *ATXN* expression was measured by RT-qPCR. Values are plotted in individual heatmap for each gene. For visualization, the qPCR values were scaled to zero mean and unit variance (relative values from 4 to - 4). Brain regions were grouped as followed: Basal ganglia and related structures (Amygdala, Caudate, Pallidum, Thalamus), Blood, Cerebellum, Fetus, Cortex (Frontal cortex, Motor cortex, Visual cortex), Brainstem (Midbrain, Substantia nigra, Pons, Olive, Medulla oblongata). SCA1: n = 1 (cerebellum: n = 3), SCA2: n = 1, SCA3: n = 3, SCA7: n = 2. White fields: to no data available.

Figure S5

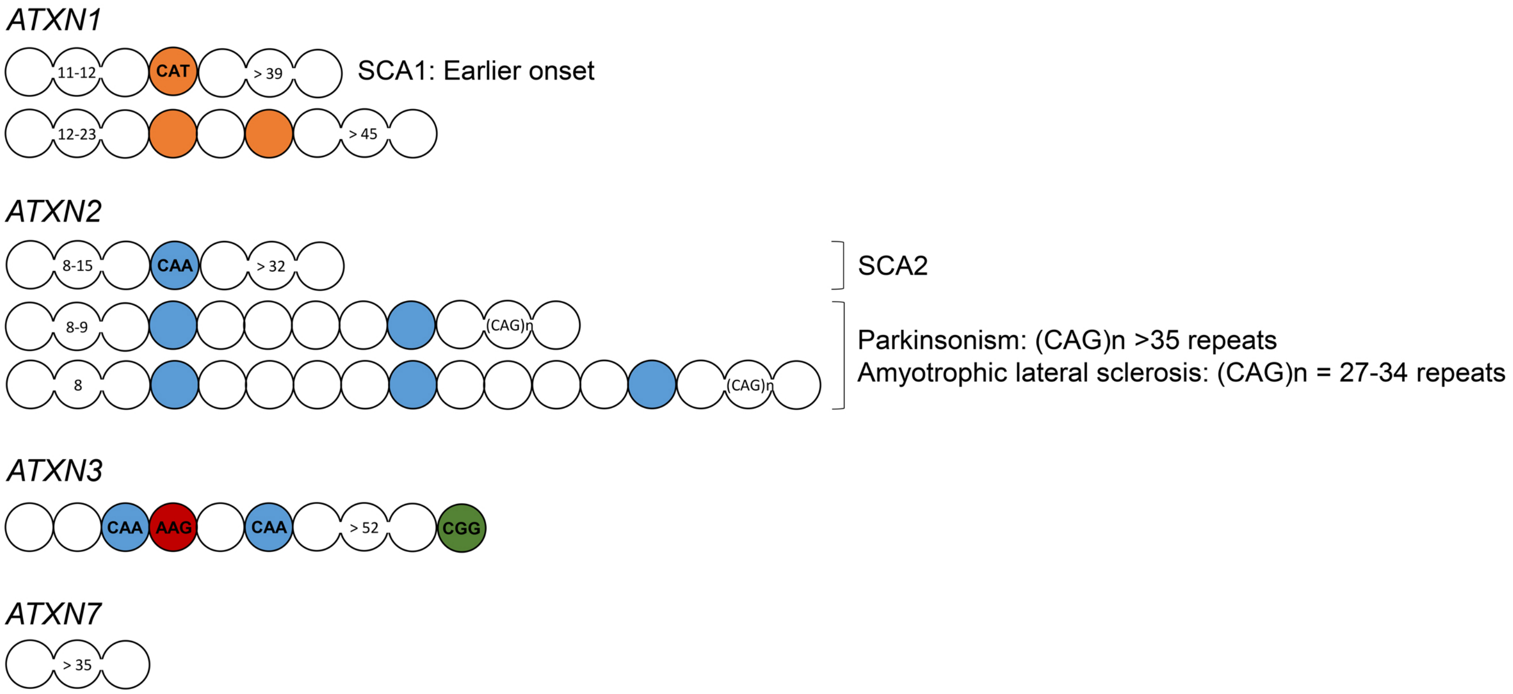

ATXN1
11-12
CAT
> 39
SCA1: Earlier onset
12-23
> 45
ATXN2
8-15
CAA
> 32
SCA2
8-9
(CAG)n
8
(CAG)n
Parkinsonism: (CAG)n >35 repeats
Amyotrophic lateral sclerosis: (CAG)n = 27-34 repeats
ATXN3
CAA
AAG
CAA
> 52
CGG
ATXN7
> 35

Figure S5. Structure of pathological CAG repeat in *ATXN1*, *ATXN2*, *ATXN3*, *ATXN7*. CAT interruption are in orange, CAA in blue, AAG in red and CGG in green. White circles represent CAG triplets, with the number of repeat for pathological manifestation written inside the circles.

Table S1 – Cohort description, longitudinal group

		CAG not expanded	Expansion rate (EI/years)	Age at death	Age at onset
**SCA1**	n	30	30	13	28
	mean	30,3	0,026	49,1	36,0
	sd	1,8	0,021	13,7	11,1
	min	26,0	- 0,016	27,5	14,0
	max	36,0	0,093	69,9	53,0
**SCA2**	n	50	50	11	41
	mean	22,3	0,013	48,5	34,2
	sd	1,0	0,016	15,7	10,8
	min	22,0	- 0,037	26,0	12,0
	max	28,0	0,054	72,7	65,0
**SCA3**	n	74	74	22	71
	mean	21,7	0,010	60,5	40,3
	sd	4,5	0,016	11,7	10,0
	min	14,0	- 0,075	38,3	16,0
	max	35,0	0,085	79,4	65,0
**SCA7**	n	30	30	6	21
	mean	10,3	0,084	55,2	27,3
	sd	1,1	0,133	8,0	10,0
	min	9,0	- 0,151	46,1	5,0
	max	13,0	0,671	67,6	47,0

Table S1. Descriptive data on the longitudinal cohort. n: number of individuals, SD: Standard deviation, Min: Minimum observed value, Max: Maximum observed value. Expansion rate: rate of expansion per year.

Table S2 – Cohort description, longitudinal group SCA1

		CAG	EI	Age	Disease duration	SARA	Severity	Disability stage
First visit	n	30	30	30	28	14	11	21
	mean	48,2	0,99	39,2	3,6	7,86	2,07	2,4
	sd	6,3	0,23	13,2	8,9	4,71	1,57	1,4
	min	40,0	0,46	17,7	-17,6	0,50	-0,79	0,0
	max	64,0	1,43	60,7	29,6	14,50	5,33	6,0
Second visit	n	30	30	30	28	19	17	21
	mean	48,3	1,13	44,7	9,3	17,53	2,31	4,0
	sd	6,2	0,26	13,7	8,3	8,80	0,86	1,8
	min	40,0	0,60	23,5	-5,4	0,00	0,71	0,0
	max	64,0	1,72	68,8	36,9	32,00	4,16	6,0
Third visit	n	12	12	12	10	9	7	9
	mean	48,3	1,25	45,3	10,1	16,56	2,47	3,4
	sd	5,0	0,29	9,1	5,5	11,49	1,34	2,2
	min	42,0	0,78	33,3	2,7	2,00	0,69	0,0
	max	59,0	1,78	63,1	23,1	34,50	4,66	6,0
Fourth visit	n	4	4	4	3	4	3	4
	mean	46,8	1,20	49,5	16,3	17,88	1,61	3,5
	sd	3,7	0,17	10,7	7,1	15,20	1,07	2,5
	min	42,0	1,01	38,3	10,1	3,00	0,54	1,0
	max	51,0	1,38	64,1	24,1	39,00	2,67	7,0
Fifth visit	n	2	2	2	1	2	1	2
	mean	47,5	1,27	45,0	11,8	11,50	1,78	2,5
	sd	0,7	0,31	8,1	NA	13,44	NA	3,5
	min	47,0	1,05	39,3	11,8	2,00	1,78	0,0
	max	48,0	1,48	50,8	11,8	21,00	1,78	5,0

Table S3 – Cohort description, longitudinal group SCA2

		CAG	EI	Age	Disease duration	SARA	Severity	Disability stage
First visit	n	50	50	50	41	25	18	36
	mean	39,7	0,61	40,0	6,6	7,76	1,75	2,6
	sd	2,9	0,21	13,0	7,3	5,23	1,20	1,1
	min	35,0	0,26	18,2	-13,3	0,00	-0,90	1,0
	max	49,0	1,22	68,9	21,8	16,00	4,14	6,0
Second visit	n	50	50	50	41	37	29	29
	mean	39,7	0,69	45,5	12,3	13,82	1,62	3,2
	sd	2,9	0,26	13,4	8,4	10,51	0,93	1,9
	min	35,0	0,28	19,3	1,1	0,00	0,00	0,0
	max	49,0	1,40	76,5	31,5	40,00	4,23	7,0
Third visit	n	21	21	19	16	17	14	13
	mean	40,0	0,70	42,1	12,0	14,24	1,35	3,3
	sd	3,2	0,27	8,7	5,9	9,77	0,42	1,9
	min	35,0	0,29	29,5	4,7	0,00	0,68	1,0
	max	47,0	1,20	63,2	27,0	34,00	1,92	6,0
Fourth visit	n	4	4	4	4	4	4	4
	mean	40,3	0,68	44,4	12,9	14,75	1,08	3,3
	sd	5,0	0,39	13,0	3,7	12,06	0,62	1,9
	min	35,0	0,29	34,9	8,0	5,50	0,41	2,0
	max	47,0	1,20	63,6	16,9	32,00	1,90	6,0
Fifth visit	n	4	3	3	3	3	3	2
	mean	40,3	0,53	49,5	13,5	10,00	0,77	3,0
	sd	5,0	0,24	13,2	4,1	4,58	0,31	0,0
	min	35,0	0,30	40,0	9,0	6,00	0,41	3,0
	max	47,0	0,77	64,6	16,9	15,00	1,00	3,0
Sixth visit	n	1	1	1	1	1	1	1
	mean	40,0	0,99	53,5	26,5	23,00	0,87	4,0
	sd	NA	NA	NA	NA	NA	NA	NA
	min	40,0	0,99	53,5	26,5	23,00	0,87	4,0
	max	40,0	0,99	53,5	26,5	23,00	0,87	4,0
Seventh visit	n	1	1	1	1	1,00	1,00	1
	mean	40,0	1,06	54,6	27,6	27,00	0,98	5,0
	sd	NA	NA	NA	NA	NA	NA	NA
	min	40,0	1,06	54,6	27,6	27,00	0,98	5,0
	max	40,0	1,06	54,6	27,6	27,00	0,98	5,0
Eighth visit	n	1	1	1	1	1	1	1
	mean	40,0	1,03	55,7	28,7	25,00	0,87	5,0
	sd	NA	NA	NA	NA	NA	NA	NA
	min	40,0	1,03	55,7	28,7	25,00	0,87	5,0
	max	40,0	1,03	55,7	28,7	25,00	0,87	5,0

Table S4 – Cohort description, longitudinal group SCA3

		CAG	EI	Age	Disease duration	SARA	Severity	Disability stage
First visit	n	74	74	74	71	32	29	54
	mean	71,5	0,58	43,7	4,1	7,67	1,37	2,3
	sd	4,4	0,14	12,7	9,8	6,26	1,47	1,3
	min	49,0	0,30	17,0	- 23,1	0,00	- 1,63	0,0
	max	78,0	0,91	74,5	26,3	26,00	5,37	6,0
Second visit	n	74	74	74	71	51	47	58
	mean	71,5	0,65	51,2	11,6	12,84	1,32	3,5
	sd	4,4	0,15	11,6	8,1	8,41	0,81	1,6
	min	49,0	0,38	28,3	- 14,7	0,00	0,00	1,0
	max	78,0	1,06	76,4	35,2	39,00	3,66	7,0
Third visit	n	30	30	30	29	25	22	23
	mean	70,8	0,64	53,3	13,2	13,92	1,36	3,4
	sd	5,1	0,13	11,0	8,1	8,99	0,79	1,8
	min	49,0	0,47	34,3	1,2	2,00	0,30	1,0
	max	77,0	0,90	77,4	29,4	31,00	3,79	6,0
Fourth visit	n	12	10	10	9	9	8	8
	mean	70,2	0,66	52,6	12,2	8,67	1,14	2,1
	sd	2,7	0,13	9,2	8,6	6,37	0,57	1,5
	min	65,0	0,48	37,4	5,3	0,00	0,28	0,0
	max	73,0	0,93	68,3	31,5	23,00	1,90	5,0
Fifth visit	n	3	4	3	3	3	3	3
	mean	70,3	0,57	52,4	8,1	7,33	0,92	1,3
	sd	3,8	0,13	6,8	0,4	3,33	0,45	0,6
	min	66,0	0,49	46,0	7,8	4,50	0,53	1,0
	max	73,0	0,76	59,5	8,5	11,00	1,42	2,0
Sixth visit	n	1	1	1	1	1	1	1
	mean	73,0	0,65	52,6	8,6	11,00	1,28	2,0
	sd	NA	NA	NA	NA	NA	NA	NA
	min	73,0	0,65	52,6	8,6	11,00	1,28	2,0
	max	73,0	0,65	52,6	8,6	11,00	1,28	2,0

Table S5 – Cohort description, longitudinal group SCA7

		CAG	EI	Age	Disease duration	SARA	Severity	Disability stage
First visit	n	30	30	30	21	19	12	24
	mean	44,1	1,85	35,9	8,8	6,76	1,78	2,0
	sd	5,5	0,73	10,0	5,7	6,20	1,39	1,2
	min	38,0	0,45	18,2	1,1	0,00	0,92	0,0
	max	63,0	2,91	58,9	25,6	15,00	5,97	4,0
Second visit	n	30	30	30	21	26	18	24
	mean	44,6	2,24	42,1	15,0	13,46	1,39	3,5
	sd	5,6	0,93	10,6	7,2	11,57	0,65	2,2
	min	38,0	0,58	19,1	2,7	0,00	0,38	0,0
	max	63,0	3,80	64,3	27,8	34,00	3,37	6,0
Third visit	n	12	12	12	7	11	6	8
	mean	41,6	2,22	50,9	17,2	13,41	1,19	3,9
	sd	2,9	1,05	8,4	6,7	13,06	0,27	2,4
	min	38,0	0,75	33,7	4,7	0,00	0,79	1,0
	max	45,0	3,81	65,5	25,6	33,00	1,61	6,0
Fourth visit	n	4	4	4	2	3	1	1
	mean	41,0	1,63	51,5	11,9	5,50	0,85	1,0
	sd	3,6	0,77	7,4	4,1	6,14	NA	NA
	min	38,0	0,95	43,9	8,9	1,00	0,85	1,0
	max	45,0	2,35	61,5	14,8	12,50	0,85	1,0

Table S2-S5. Descriptive data on the longitudinal cohort. (S2) SCA1 cohort, (S3) SCA2 cohort, (S4) SCA3 cohort, (S5) SCA7 cohort. CAG: reference CAG at diagnosis, EI: Expansion index, SARA: Scale for the Assessment and Rating of Ataxia, Severity: SARA score corrected by disease duration. Disability score: 0 (no functional handicap), 1 (no functional handicap but signs at examination), 2 (mild, able to run, unlimited walking), 3 (moderate, unable to run, limited walking without aid), 4 (severe, walking with one stick), 5 (walking with two sticks), 6 (unable to walk, requiring a wheelchair), 7 (confined to a bed). n: number of individuals, SD: Standard deviation, Min: Minimum observed value, Max: Maximum observed value.

Table S6. Atrophy, neuron loss and white matter degeneration seen at macroscopic and microscopic examination of post-mortem.

**Patient ID**	**1**	**2**	**3**	**4**	**5**	**6**	**7**	**8**	**9**	**10**
Age at death (y)	42	57	50	35	68	42	56	72	55	56
Sex	M	F	M	M	F	M	F	F	M	M
Diagnosis	SCA1	SCA1	SCA1	SCA2	SCA3	SCA3	SCA3	SCA3	SCA7	SCA7
CAG repeats	55	49	49	47	73	78	74	70	42	42
Weight (g)	1040	1142	1122	1068	1078	NA	1180	1196	NA	1324
FF / NF	FF	FF	FF	NF	FF	NA	NF	FF	NA	NF
Macroscopic examination (atrophy)										
Neocortex	No	No	No	No	No	na	No	No	na	No
Striatum	No	No	No	No	No	na	No	No	na	No
Pallidum	No	Yes	No	No	Yes	na	No	No	na	No
Thalamus	No	No	No	No	No	na	No	No	na	No
STN	No	Yes	No	No	Yes	na	No	No	na	No
Amygdala	No	No	No	No	No	na	No	No	na	No
Hippocampus	No	No	No	No	No	na	No	No	na	No
SN (depigmentation)	No	No	No	Yes	Yes	na	Yes	Yes	na	No
Pons	Yes	Yes	Yes	Yes	Yes	na	Yes	Yes	na	Yes
Inf olive	Yes	na	Yes	na	No	na	No	No	na	Yes
Cerebellum	Yes	Yes	Yes	Yes	Yes	na	Yes	No	na	Yes
Dentate gyrus	Yes	Yes	Yes	No	Yes	na	na	No	na	na
Cerebellar peduncles	na	Yes	na	na	Yes	na	Yes	No	na	na
Cerebellum (WM)	na	na	na	Yes	na	na	na	No	na	na
Spinal cord	na	na	na	Yes	na	na	Yes	na	na	na
Microscopic examination (neuron loss or white matter degeneration)										
Neocotex	0	0	0	0	0	na	0	0	na	0
Striatum	0	0	0	0	0	na	na	0	na	na
Pallidum	na	3	0	0	3	na	0	na	na	na
Thalamus	0	0	0	0	0	na	0	0	na	na
STN	0	na	0	na	3	na	1	0	na	na

Amygdala		na	0	0	0	0	na	na	0	na	na
Hippocampus		0	0	0	0	0	na	0	2*	na	na
SN		0	1	1	3	3	na	2	2	na	0
Pons	*LC*	0	0	0	1	0	na	1	0	na	0
	PN	2	3	2	3	3	na	2	2	na	na
	TF	3	3	2	3	3	na	2	0	na	na
	Sup CP	3	2	3	0	0	na	1	0	na	na
Med Oblon	*Inf olive*	3	3	3	3	1	na	0	0	na	3
Cerebellum	*Purkinje cells*	1	1	1	3	1	na	0	1	na	3
	Dentate n	3	3	3	1	2	na	2	2	na	3
	WM	1	2	3	3	1	na	1	1	na	na
Spinal cord	*Post tract*	3	na	na	3	na	na	3	na	na	na
	SPC T	2	na	na	0	na	na	2	na	na	na
	MN	0	na	na	0	na	na	0	na	na	na
IHC (p62/ubiquitin/1C2)		yes	yes	yes	na	yes	na	yes	na	na	yes

FF: weight of brain when fixed in formalin; Inf olive: inferior olive; LC: *locus cœruleus*; Med Oblon: medulla oblongata; MN: motor neurons; na: not available; NF: weight of non-fixed brain; PN: pontine nuclei; SN: *substantia nigra*; SPC T: spinocerebellar tract; STN: subthalamic nucleus; Sup CP: superior cerebellar peduncle; TF: Transverse fibers; WM: white matter; y: years.
*: hippocampal neuron loss secondary to an old hemorrhagic infarct.

Table S6. Atrophy, neuron loss and white matter degeneration seen at macroscopic and microscopic examination of post-mortem. FF: weight of brain when fixed in formalin; Inf olive: inferior olive; LC: locus cœruleus; Med Oblon: medulla oblongata; MN: motor neurons; na: not available; NF: weight of non-fixed brain; PN: pontine nuclei; SN: substantia nigra; SPC T: spinocerebellar tract; STN: subthalamic nucleus; Sup CP: superior cerebellar peduncle; TF: Transverse fibers; WM: white matter; y: years. At the microscopic level, atrophy was evaluated from 0 (no degeneration) to 3 (important degeneration). *: hippocampal neuron loss secondary to an old hemorrhagic infarct. Na: data not available

Supplemental Methods

Primers sequence - determination of CAG length by PCR

	Forward sequence	*Reverse sequence*
ATXN1	[DGFO]CTGGCCAACATGGGCAGTCTGAG	GCGGAGAACTGGAAATGTGGACGTA
ATXN2	[HEX]GCCCCTCACCATGTCGCTGAA	GGGCTTGCGGACATTGGCAG
ATXN3	[6FAM]CGAGTTCCAGTGACTACTTTGATTCGTGAAACA	TGAACTGGTGGCTGGCCTTTCACAT
ATXN7	[HEX]CATTGTAGGAGCGGAAAGAATGTCGGAG	CCACGACTGTCCCAGCATCACTTCA

Primers sequences - qPCR

	Forward sequence	*Reverse sequence*
ATXN1	TCGGTGGAGCTTGGTTTACAA	GGGAGGACCCAATGAACTGG
ATXN2	TTGATGCCGCACATGAGAAAA	CGCCATTCACTTTAGCACTGAT
ATXN3	TGTGCTCAACATTGCCTGAAT	GCTGCTGTAAAAACGTGCGATA
ATXN7	TAGTCAAGCCTGGCCTTAACT	GTGTCGAGATCAATAACCCCAC
PPIA	CCCACCGTGTTCTTCGACATT	GGACCCGTATGCTTTAGGATGA
MLH1	CAACAAGTCTGACCTCGTCTTC	CCGGGAATCTGTACGAACCAT
FAN1	AGCAGAAGATCAGTCCCTACTT	TGCTAGGCTTCCCAAACAAATG
MSH3	TGTGAATCCCCTAATCAAGCTGG	GCACAGAAGATAGCTGGTAGAAG
MSH2	CACTGTCTGCGGTAATCAAGT	CTCTGACTGCTGCAATATCCAAT
PMS2	CAATGGATGTGGGGTAGAAGAAG	GTTAGGTCGGCAAACTCTTGAAT
PMS1	ACTTACGGTTTTCGTGGAGAAG	AGCAGCCGTTCTTGTTGTAAT
LIG1	ACAGTTCCCCATCAGGGATTC	CTCTGTGAGGCTTTCTTTCGG
MLH3	TCTCTCACTCATGCACCCTTC	TCGGGAACATACGTCTTTGGT
MSH6	CCAAGGCGAAGAACCTCAAC	ACCAGGGGTAACCCTCCATC
